# Effects of prior heavy exercise on deoxygenation in deep versus superficial muscle regions during subsequent heavy exercise in adult males

**DOI:** 10.14814/phy2.70852

**Published:** 2026-04-10

**Authors:** Weerapong Chidnok, Dai Okushima, Richie P. Goulding, David C. Poole, Thomas J. Barstow, Andrew M. Jones, Pieter‐Jan Marent, Narihiko Kondo, Shunsaku Koga

**Affiliations:** ^1^ Exercise and Rehabilitation Sciences (ERS) Research Unit, Department of Physical Therapy, Faculty of Allied Health Sciences Naresuan University Phitsanulok Thailand; ^2^ Nagaoka University of Technology Nagaoka Japan; ^3^ Laboratory for Myology, Department of Human Movement Sciences, Faculty of Behavioral and Human Movement Sciences, Vrije Universiteit Amsterdam Amsterdam Movement Sciences Amsterdam the Netherlands; ^4^ School of Health Sciences and Department of Anatomy and Physiology Kansas State University Manhattan Kansas USA; ^5^ Department of Public Health and Sport Sciences, Faculty of Health and Life Sciences University of Exeter Medical School, St. Luke’s Campus Exeter UK; ^6^ Department of Movement Sciences KU Leuven Leuven Belgium; ^7^ Applied Human Physiology Laboratory Kobe University Kobe Japan; ^8^ Applied Physiological Laboratory Kobe Design University Kobe Japan

**Keywords:** deep and superficial muscles, muscle deoxygenation, oxygen utilization, priming exercise, time‐resolved near‐infrared spectroscopy

## Abstract

We assessed the effects of prior heavy‐intensity‐exercise (PHE) on microvascular deoxygenation in deep *vastus lateralis* muscle (VLd) compared with the superficial VL (VLs). Thirteen subjects completed two 6‐min bouts of heavy exercise, separated by 6‐min unloaded cycling. Using time‐resolved near‐infrared spectroscopy, absolute concentrations of deoxy‐ and total[Hb + Mb] were assessed simultaneously in VLs and VLd. PHE raised the V̇O_2_ baseline and attenuated the slow component (V̇O_2SC_) during subsequent exercise. In both muscle regions, PHE reduced the deoxy[Hb + Mb] baseline and increased the amplitude, such that end‐exercise deoxy[Hb + Mb] did not differ between muscle regions. Changes in the baseline and amplitude of deoxy[Hb + Mb] induced by PHE were significantly smaller in VLd versus VLs. The primary component time constant of the deoxy[Hb + Mb] kinetics in VLd was greater than that for VLs. The end‐exercise total[Hb + Mb] in VLd was lower than that in VLs in both bouts. Despite site‐dependent differences in PHE‐induced deoxy[Hb + Mb] alterations, neither these changes nor total[Hb + Mb] correlated with V̇O_2SC_ reduction in VLs or VLd. In conclusion, these findings suggest that PHE has less effect on microvascular Q̇O_2_‐to‐V̇O_2_ matching during heavy exercise in VLd versus VLs. Moreover, PHE‐induced changes in microvascular oxygen transport were unrelated to the PHE‐induced speeding of pulmonary V̇O_2_ kinetics.

## Graphical abstract

Graphical summary of the effect of priming exercise on superficial vs. deep muscle deoxygenation.

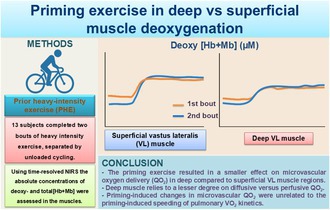

## 1 INTRODUCTION

Prior heavy‐intensity exercise (PHE) speeds the overall pulmonary oxygen uptake (V̇O_2_) response to subsequent heavy or severe exercise (Gerbino et al., 1996) and enhances exercise performance (Bailey et al., 2009; Burnley et al., 2011; Goulding et al., 2023; Jones et al., 2003; Macdougall et al., 2025). The mechanisms underpinning this effect have received much attention (Goulding et al., 2023; Spencer et al., 2012). Specifically, the priming effect has been variously attributed to (1) enhanced mitochondrial enzyme activity (Behnke et al., 2002; Christensen et al., 2016; DeLorey et al., 2007; Gandra et al., 2012; Gurd et al., 2006; Hogan, 2001), (2) increased bulk and local O_2_ delivery (Q̇O_2_) (DeLorey et al., 2007; Fukuoka et al., 2015; Gerbino et al., 1996; Hernandez et al., 2010; MacDonald et al., 2001), (3) a rightward shift of the oxyhemoglobin dissociation curve (Fukuoka et al., 2015; Gerbino et al., 1996; Jones et al., 2011; Poole et al., 1991), and (4) altered motor unit recruitment patterns (Burnley et al., 2000; Burnley & Baker, 2005). However, given the marked heterogeneity of muscle metabolism and deoxygenation within and between muscle groups, whether these factors are consistent within a given individual in different muscle regions has not been addressed.

During heavy intensity exercise (i.e., above the gas exchange threshold, GET), the fundamental V̇O_2_ response is supplemented by an additional slow component of the V̇O_2_ kinetics (V̇O_2SC_). This V̇O_2SC_ elevates the oxygen cost of work compared to below the GET, is accompanied by an elevated blood [lactate], and reduces work efficiency and exercise tolerance (Goulding et al., 2021, Okushima, et al., 2021; Grassi et al., 2015; Jones et al., 2011). Although early studies suggested that PHE resulted in a speeding of the primary component kinetics (i.e. a reduced time constant of the primary response, τ_p_), it was later shown that this only occurs when τ_p_ is large (i.e., V̇O_2_ kinetics are slow) in the control (i.e., non‐primed) condition (Goulding et al., 2023). Instead, in young healthy individuals, the priming effect typically occurs via reduction in the V̇O_2SC_ amplitude and does not alter the primary component V̇O_2_ kinetics (Burnley et al., 2000; Koppo & Bouckaert, 2001, 2002). It is, at present, not known how changes in each of these phases (especially the reduced V̇O_2SC_) relate to modifications in local intramuscular oxygenation patterns following PHE.

In humans, skeletal muscle is structurally (fiber type and vascular density) and functionally (fiber activation, vascular blood flow and metabolic control) heterogeneous and these latter differences become exaggerated during exercise (Heinonen et al., 2015; Johnson et al., 1973; Kalliokoski et al., 2001; Kalliokoski et al., 2004; Koga et al., 2014; Poole et al., 2020). Further, the quadriceps femoris muscle's deeper regions evince a greater type I fiber population which is more highly vascularized (McDonough et al., 2005) and thus are likely to have greater Q̇O_2_‐to‐V̇O_2_ ratios. This observation is considered to explain the slower deoxygenation kinetics of deep versus superficial muscle during exercise, the latter likely being comprised of a greater proportion of type II fibers (Koga et al., 2015). It remains to be determined whether PHE‐induced changes in deep muscle tissue red blood cell distribution or deoxygenation affect pulmonary V̇O_2_ kinetics.

Most studies assessing the effects of PHE on muscle deoxygenation during exercise employed single‐site, continuous‐wave near‐infrared spectroscopy (CW‐NIRS) measurements of superficial muscle (Boyes et al., 2019; Cleland et al., 2012; DeLorey et al., 2007; Jones et al., 2006; Layec et al., 2009; Prieur et al., 2010; Saitoh et al., 2009). However, the local muscle deoxygenation dynamics may have been related to intra‐ and intersubject variability in unmeasured optical factors such as path length and scattering coefficients inherent in CW‐NIRS technology (Chin et al., 2011; Endo et al., 2021; Ferreira et al., 2007). In contrast, time‐resolved (TR‐) NIRS enables the measurement of the path length and scattering coefficients and thus the determination of absolute values of deoxy‐ and total[Hb + Mb], and therefore constitutes a more valid measure of fractional O_2_ extraction when compared to conventional CW‐NIRS (e.g., (Goulding et al., 2020)). Moreover, the ability to sample superficial and deep muscles with TR‐NIRS provides greater insight into the mechanisms underpinning changes in O_2_ transport and utilization afforded by PHE. In this regard, the relationship between muscle deoxygenation and pulmonary V̇O_2_ kinetics is unclear, and this relationship is further complicated by intra and inter muscular heterogeneity. For example, priming exercise usually slows muscle deoxy[Hb + Mb] kinetics and speeds overall V̇O_2_ kinetics (Goulding et al., 2020). In contrast, both exercise training and PHE performed in individuals with type I diabetes speed muscle deoxy[Hb + Mb] kinetics and V̇O_2_ kinetics concomitantly (Goulding et al., 2020). Clearly, there is a complex, context‐dependent relationship between local muscle deoxygenation and whole‐body V̇O_2_ kinetics compelling further study. The significant degree of intra‐ and inter‐muscular heterogeneity in muscle deoxy‐[Hb + Mb] kinetics (Koga et al., 2007), that is, larger time constant and lower baseline values in deep versus superficial muscle (Goulding et al., 2021; Koga et al., 2017; Okushima et al., 2015), adds a further layer of complexity to this relationship. To clarify the contribution of changes in muscle deoxygenation to the whole‐body effects of priming exercise, and to illuminate the relationship between alterations in deep muscle deoxygenation and whole‐body V̇O_2_ responses, further studies of PHE are warranted.

Herein we investigated PHE effects on superficial vs. deep muscle oxygenation during heavy exercise by resolving absolute deoxy‐ and total[Hb + Mb] response profiles by TR‐NIRS. We hypothesized that PHE would reduce baseline deoxy[Hb + Mb] and slow the deoxygenation kinetics to a greater extent in the superficial vastus lateralis (VLs) than in the deep region (VLd), due to the higher oxidative capacity and vascularization of VLd which may blunt the impact of PHE. Consequently, we expected a reduced disparity in deoxygenation profiles between VLs and VLd after PHE. Further, we hypothesized that individuals exhibiting a greater PHE‐induced speeding of pulmonary V̇O_2_ kinetics (i.e., reduced *τ*_*p*_) would also show a more pronounced change in muscle deoxy[Hb + Mb] kinetics, particularly in VLs where the priming effect is presumed to be more impactful.

## 2 MATERIALS AND METHODS

### 2.1 Ethical approval

The study was approved by the Kobe Design University Research Ethics Committee (2016‐1, 2018‐1), in accordance with the declaration of Helsinki. Procedures and risks were explained to each subject who gave written informed consent to participate in this study. The subjects were all recreationally active and were familiar with the experimental procedures used in the study.

### 2.2 Participants

Thirteen males (mean ± SD: age 23 ± 4 years, stature 1.73 ± 0.09 m, body mass 65.0 ± 10.8 kg) participated in this study. Participants were non‐smokers with no known musculoskeletal, respiratory, cardiovascular, or metabolic conditions, and none of them took any medications that might influence cardiorespiratory or metabolic responses to exercise.

### 2.3 Experimental protocol

On test days, subjects were instructed to report to the laboratory in a rested state, having abstained from alcohol and strenuous exercise within the previous 24 h, and from food and caffeine for the preceding 3 h. The experiment was conducted in an environmental chamber (FLC‐2700S; Fuji Medical Science, Chiba, Japan) maintained at an ambient temperature of 24°C and relative humidity of 50%. The subjects visited the laboratory on four occasions over a 4‐week period to perform exercise tests on an electronically‐braked cycle ergometer (XL‐75III; Combi, Tokyo, Japan) in the upright position. On the first visit, subjects completed a ramp incremental exercise test to determine peak V̇O_2_ (V̇O_2peak_) and the gas exchange threshold (GET). During the second visit, subjects completed a 6 min heavy‐intensity cycling exercise test to confirm the presence of the V̇O_2SC_ which is emblematic of heavy‐intensity exercise. Following these tests, subjects completed two separate visits whereby participants were required to perform two repeated 6‐min bouts of heavy‐intensity exercise tests, separated by 6 min of unloaded cycling. Test days were separated by at least 48 h.

### 2.4 Exercise testing

#### 2.4.1 Ramp incremental exercise test

The test consisted of 2 min of rest, followed by 4 min of pedaling at 20 W, followed by a continuous ramped increase in work rate of 20 W min^−1^ until the subject was unable to continue. The subjects cycled at a cadence of 60 rpm and tests were terminated when the required pedal rate could no longer be maintained (i.e., fell by >5 rpm). Peak power output was recorded and V̇O_2peak_ was defined as the highest 30 s mean value recorded. The GET was determined from a cluster of measures including: (1) the first disproportionate increase in carbon dioxide output (V̇CO_2_) from visual inspection of individual plots of V̇CO_2_ versus V̇O_2_; (2) an increase in V̇E/V̇O_2_ (V̇E, expiratory ventilation) with no increase in V̇E/V̇CO_2_; (3) an increase in end‐tidal O_2_ tension with no fall in end‐tidal CO_2_ tension (Beaver et al., 1986).

#### 2.4.2 Heavy intensity constant power output exercise tests

Square‐wave exercise transition tests were conducted on separate days. The power outputs for heavy exercise were calculated as a work rate halfway between the V̇O_2_ at GET (V̇O_2GET_) and V̇O_2peak_ (i.e., V̇O_2_ at 50%Δ = V̇O_2GET_ + 0.5 (V̇O_2peak_‐V̇O_2GET_), based on the V̇O_2_/work rate with account taken of the lag in V̇O_2_ relative to the work rate during ramp exercise (Boone et al., 2008; Goulding et al., 2017). The PHE protocol consisted of 2 min of rest, 4 min of baseline pedaling at 20 W, followed by two 6‐min bouts of heavy intensity exercise separated by 6 min of 20 W exercise at a pedal frequency of 60 rpm. This protocol was performed twice for each subject at the same time of day ±2 h.

### 2.5 Measurements

#### 2.5.1 Pulmonary V̇O_2_

Throughout all tests, pulmonary V̇O_2_ was measured by a breath‐by‐breath gas exchange measurement system (Aeromonitor AE‐300S; Minato‐Medical, Osaka, Japan). This system was calibrated according to the manufacturer's recommendation before each exercise test. Subjects breathed through a low‐resistance, hot‐wire flow meter for measurement of inspiratory and expiratory flows and volumes. Inspired and expired oxygen and carbon dioxide concentrations were continuously sampled from the mouth and measured by this system. Gas volume and concentration signals were time aligned during gas analysis to account for the time lag between the signals.

#### 2.5.2 Muscle deoxygenation and [hematocrit]

Absolute muscle oxy‐ (oxy[Hb + Mb]), deoxy‐ (deoxy[Hb + Mb]) and total hemoglobin plus myoglobin concentration (total[Hb + Mb]) were measured by two TR‐NIRS machines (TRS‐20 [superficial muscle] and TRS‐20SD [deep muscle]; Hamamatsu Photonics K.K., Hamamatsu, Japan). The probes were placed on the surface of the VL muscle to measure both the superficial and deep regions of the VL (VLs and VLd). The interoptode spacing (OS) between emitter and receiver for superficial and deep muscle were 3 and 6 cm, respectively. The centers of the OS on the superficial and deep muscles were located 9.7 and 19.3 cm proximal to the upper border of the patella for all participants, respectively. The measurement sites on the quadriceps were shaved and cleaned with alcohol before each experiment. The method for mounting the TR‐NIRS optodes was the same as Okushima et al. (2015). The measurement principles, reliability and validity of the TRS‐20 and TRS‐20SD system has been described in detail in previous studies (Hamaoka et al., 2000; Ijichi et al., 2005; Koga et al., 2015).

#### 2.5.3 Adipose tissues thickness (ATT)

The ATT (in millimeters) was determined with the subject standing in an anatomical position on the day of the first heavy intensity constant exercise test. The depth of muscle and adipose tissue thickness under the NIRS optode was measured by Doppler ultrasound (Logiq400; GE‐Yokogawa Medical Systems, Tokyo, Japan). The ultrasonographic images were collected with care so as to prevent compression of muscle or other tissues under the probe.

### 2.6 Data analysis

To account for the influence of subcutaneous ATT on the NIRS signals, a correction coefficient was calculated based on the linear relationship between total[Hb + Mb] and ATT as described in previous studies (Koga et al., 2015; Okushima et al., 2015).

The breath‐by‐breath V̇O_2_ data were initially examined to exclude errant breaths caused by coughing, swallowing, sighing, etc., and those values lying >3 standard deviations from the local mean were removed. The data were subsequently linearly interpolated to provide second‐by‐second values. For each subject, the data sets for the two visits were time‐aligned and ensemble averaged. The response curve of V̇O_2_ was fit by a tri‐exponential function (Equation 1) that included amplitudes, time constants, and time delays, using nonlinear least‐squares regression techniques (Fukuba et al., 2002; Grassi et al., 2003) as described in the following equation:

(1) $$\begin{array}{c} \;H\left(t\right)=0\;\text{for}\;t<0\\ \;=1\;\text{for}\;t\geq 0\\ \dot{V}O_{2}\left(t\right)=\dot{V}O_{2\text{baseline}}+H\left(t\right)\cdot A_{c}\cdot \left(1-e^{-t/\tau_{c}}\right)\ldots \text{phase}\;I\;\left(\text{cardiovascular component}\right)\\ \;+H\left(t-{\mathrm{TD}}_{p}\right)\cdot A_{p}\cdot \left[1-e^{-\left(t-{\mathrm{TD}}_{p}\right)/\tau_{p}}\right]\ldots \text{phase}\;\mathrm{II}\;\left(\text{primary component}\right)\\ \;\;+H\left(t-{\mathrm{TD}}_{s}\right)\cdot A_{s}\cdot \left[1-e^{-\left(t-{\mathrm{TD}}_{s}\right)/\tau_{s}}\right]\ldots \text{phase}\;\mathrm{III}\;\left(\text{slow component}\right) \end{array}$$

where the subscripts *c*, *p* and *s* refer to cardiodynamic, primary and slow components, respectively; Baseline represents the mean V̇O_2_ measured over the final 60 s of baseline pedaling; A is the asymptotic amplitudes for the exponential terms; τ_p_ represents the time constant; and TD represents the time delays. Mean response time (MRT_*p*_) for the primary phase of V̇O_2_ was defined as the sum of primary component TD_*p*_ and *τ*_*p*_. The phase I V̇O_2_ at the start of phase II (i.e., at TD_*p*_) was assigned the value for that time (A_*c*_′). The physiologically relevant amplitude of the primary exponential component during phase II (A_*p*_') was defined as the sum of A_*c*_′ and A_*p*_. Because of concerns regarding the validity of using the extrapolated asymptotic value for the SC (A_*s*_) for comparisons, we used the value of the slow exponential function at the end of exercise, defined as A_s_′ [i.e., V̇O_2_ at 360 s − (V̇O_2baseline_ + A_*p*_')]. The end‐exercise V̇O_2_ was calculated as the mean value of the last 60 s during each exercise bout. The computation of best‐fit parameters was chosen by the program to minimize the sum of the squared differences between the fitted function and the observed response (*r* ^2^ = 0.96 ± 0.01, 1st bout; 0.95 ± 0.01, second bout). This approach is common and has been used in several previous studies (Koga et al., 2007; Okushima et al., 2015).

To provide information on muscle oxygenation in VLs and VLd, the absolute deoxy[Hb + Mb] data were fit from the last 60 s at 20 W exercise to 180 s of the main exercise with a monoexponential model of the form in Equation 2 to determine the time course of muscle deoxygenation:

(2) $$\begin{array}{c} \;H\left(t\right)=0\;\text{for}\;t<0\\ \;=1\;\text{for}\;t\geq 0\\ \;\text{deoxy}\left[\mathrm{Hb}+\mathrm{Mb}\right]\left(t\right)\;=\text{deoxy}{\left[\mathrm{Hb}+\mathrm{Mb}\right]}_{\text{baseline}}+H\left(t\right)\cdot A\cdot \left[1-e^{\left(-\left(t-\mathrm{TD}\right)/\tau \right)}\right] \end{array}$$

where the deoxy[Hb + Mb]_baseline_ is the 20 W exercise baseline value; A represents the asymptotic amplitudes for the exponential terms; τ represents the time constants; and TD represents the time delay from the onset of exercise to the onset of primary component of deoxy[Hb + Mb]. The TD and τ were summed (MRT) to provide an indication of the overall dynamics of the primary component (Chin et al., 2011). The computation of best‐fit parameters was chosen by the program to minimize the sum of the squared differences between the fitted function and the observed response (*r* ^2^ = 0.93 ± 0.05–0.97 ± 0.02 for VLs and *r* ^2^ = 0.74 ± 0.24–0.76 ± 0.22 for VLd). This approach is common and has been used in several previous studies (Koga et al., 2017; Okushima et al., 2015). In this study, muscle deoxygenation kinetics in the VLd for one subject were rejected due to a poor fit of the above exponential model. Moreover, we calculated the end‐exercise value of deoxy[Hb + Mb] from averaging last 60 s data and the slow component (A_6‐3_) from 30 s data immediately before 3 and 6 min of exercise during each exercise bout.

To provide information on total[Hb + Mb] concentrations, the values of baseline total[Hb + Mb] were calculated by averaging the last 60 s of 20 W exercise. The end‐exercise total[Hb + Mb] value was calculated by averaging the last 60 s value of each heavy exercise bout. The amplitude of total[Hb + Mb] was calculated as the change from baseline to 3 min value in each exercise bout. These total[Hb + Mb] parameters were calculated across all measured sites. Moreover, the slow component (A_6‐3_) was calculated from 30 s averaged data taken immediately before 3 and 6 min of exercise in each bout.

### 2.7 Statistical analysis

All data are presented as mean ± standard deviation (SD). A paired *t*‐test was used to compare the differences between the first and second bouts for V̇O_2_. Comparisons of kinetics and steady‐state concentrations of NIRS variables were analyzed by two‐way repeated measures ANOVA (time × site). The *F*‐value was examined for main effects of time and site, and for the interaction effect (time × site). A significant interaction effect (time × site) was followed up by examining the magnitude of change from 1st to 2nd bout between muscle sites. In addition, relationships between V̇O_2_/deoxy[Hb + Mb] tau in 1st bout and the change of V̇O_2_/deoxy[Hb + Mb] tau from 1st to 2nd bout were calculated using Pearson correlation coefficient. Statistical significance was accepted at *p* < 0.05. A statistical software (GraphPad Prism ver. 10.1.0, GraphPad Software, San Diego, USA) was used for each test. Effect sizes (using Cohen's *d* and *η* ^2^_p_) and statistical power (1‐β) were calculated for the pulmonary V̇O_2_ and NIRS variables.

## 3 RESULTS

The V̇O_2peak_ during ramp incremental exercise was 49.8 ± 8.6 mL min^−1^ kg^−1^ and the GET was 24.9 ± 5.8 mL min^−1^ kg^−1^. Peak power output was 275 ± 29 W with GET occurring at 120 ± 23 W. Power output for the heavy intensity exercise test was 196 ± 24 W. Adipose tissue thickness (ATT) differed significantly between the VLs and VLd sites (4.1 ± 0.9 vs. 4.7 ± 1.2 mm, *p* < 0.05). ATT and resting total[Hb + Mb] were negatively correlated (*y* = −9.69·*x* + 168, *r* ^2^ = 0.62, *p* < 0.05), with this relationship used to normalize all subsequent NIRS data.

### 3.1 Pulmonary V̇O_2_ kinetics

**FIGURE 1:**
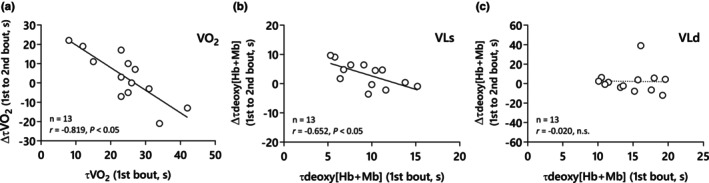
(a) The relationship between the pulmonary V̇O_2_ time constant (τ_p_) during the prior heavy‐intensity exercise (1st bout) and the changes to subsequent heavy‐intensity exercise (1st to 2nd bout). (b) Deoxy[Hb + Mb] time constant in the VLs during the prior heavy‐intensity exercise (1st bout) and the changes to subsequent heavy‐intensity exercise (1st to 2nd bout). (c) Deoxy[Hb + Mb] time constant in the VLd during the prior heavy‐intensity exercise (1st bout) and the changes to subsequent heavy‐intensity exercise (1st to 2nd bout).

The parameters of V̇O_2_ kinetics during the two heavy cycling exercise bouts are reported in Table 1. Baseline V̇O_2_ was significantly increased from prior (1st bout) to subsequent exercise (2nd bout) (*p* < 0.001, *d* = 2.19, 1‐β = 0.99) (Table 1). The TD_*p*_ and V̇O_2SC_ amplitude (A_*s*_′) were reduced in the second compared to the first bout (*p* < 0.010, *d* = 0.84, 1‐*β* = 0.79; *p* < 0.001, d = 1.50, 1‐β = 0.99) (Table 1). However, PHE did not alter the primary component time constant of pulmonary V̇O_2_ response (τ_*p*_). V̇O_2_ τ_p_ in the initial bout of exercise was negatively correlated with the change in V̇O_2_ τ_p_ between 1st and 2nd bouts (*r* = −0.819, *p* < 0.05; Figure 1a).

**TABLE 1:** The mean value of V̇O_2_ kinetic parameters during prior (1st bout) and subsequent (2nd bout) heavy cycling exercise.

	1st bout	2nd bout
BL (L/min)	0.62 ± 0.06	0.74 ± 0.08*
TDp (s)	23 ± 6	17 ± 5*
τ_p_ (s)	24 ± 9	27 ± 8
MRTp (s)	47 ± 7	44 ± 9
Ap' (L/min)	1.71 ± 0.28	1.77 ± 0.30
TDs (s)	136 ± 35	149 ± 36
As′ (slow component) (L/min)	0.31 ± 0.15	0.16 ± 0.08*
*r* ^2^	0.96 ± 0.01	0.95 ± 0.01

*Note*: Values are shown as mean ± SD.

Abbreviations: A_*p*_', amplitude of phase I and II without BL; A_*s*_′, amplitude of phase III; BL, baseline; MRTp, mean responses time of the primary component (i.e., MRT_p_ = TD_p_ + *τ*_p_); TD_*p*_, time delay of phase II; TD_s_, time delay of phase III (i.e., slow component); *τ*_*p*_, time constant of the primary component in V̇O_2_ response.

* Shows the significant exercise between exercise bouts (*p* < 0.05).

### 3.2 Deoxy*[*Hb *+* Mb*]* responses

**FIGURE 2:**
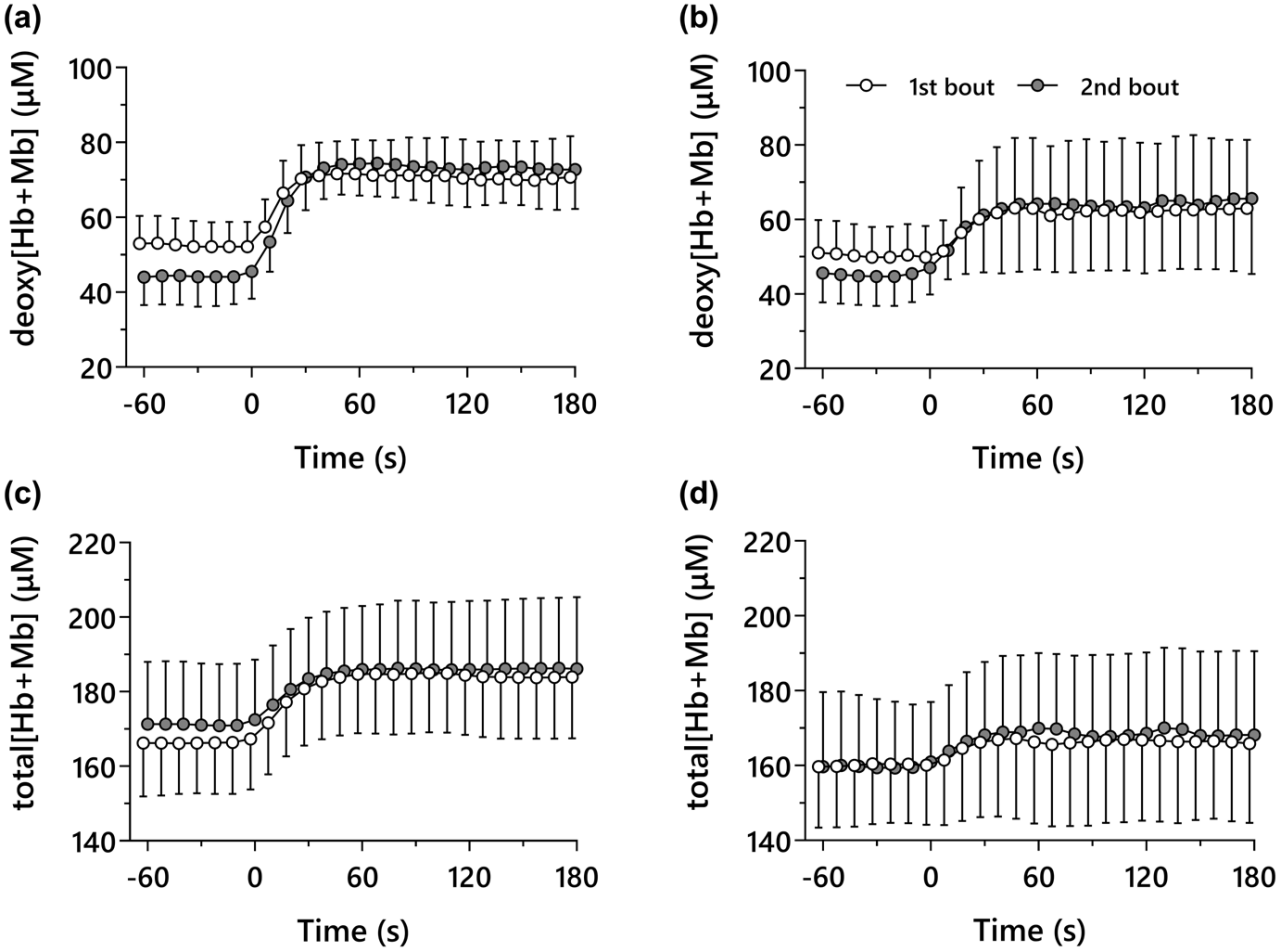
The mean responses of deoxy and total[Hb + Mb] in superficial (a, b) and deep (c, d) portion of vastus lateralis during prior (1st bout, open symbol) and subsequent (2nd bout, gray symbol) heavy cycling exercise.

**FIGURE 3:**
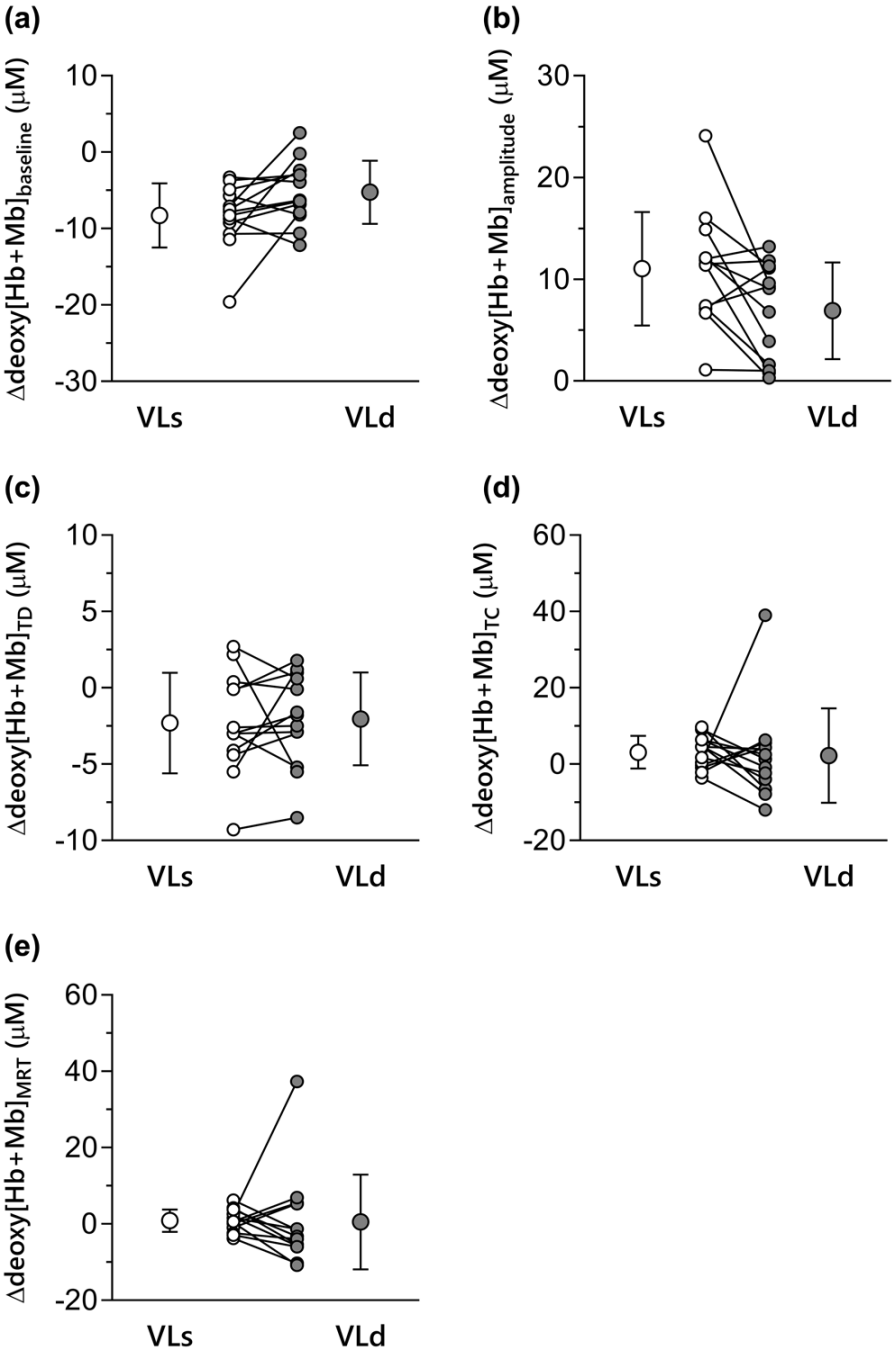
The changes (Δ) in deoxy[Hb + Mb] concentration (baseline and amplitude) and kinetics from prior to subsequent heavy‐intensity exercise in VLs (open symbol) and VLd (gray symbol). (a) Deoxy[Hb + Mb] baseline, (b) Deoxy[Hb + Mb] amplitude, (c) Deoxy[Hb + Mb] time delay, (d) Deoxy[Hb + Mb] time constant, (e) Deoxy[Hb + Mb] mean response time.

The group mean deoxy[Hb + Mb] responses are presented in Table 2, Figure 2. The changes from 1st to 2nd bout in the kinetics and steady‐state deoxy[Hb + Mb] concentrations are presented in Figure 3. The deoxy[Hb + Mb] baseline showed a significant interaction of time and site (*p* = 0.040, *η* ^2^_p_ = 0.31, 1‐β = 1.00) with a greater reduction from 1st to 2nd bout in VLs compared to VLd (*d* = 0.61, 1‐β = 0.53; Figure 3a). PHE resulted in a significant interaction of time and site on the deoxy[Hb + Mb] amplitude (*p* = 0.021, *η* ^2^_p_ = 0.37, 1‐β = 1.00) with a greater increase from 1st to 2nd bout in VLs compared to VLd (*d* = 0.73, 1‐β = 0.68; Figure 3b). Of note, the end‐exercise deoxy[Hb + Mb] concentration was not influenced by PHE in either time or muscle region. The deoxy[Hb + Mb] TD exhibited a significant main effect of time, which was significantly shortened in the 2nd bout (*p* = 0.014, *η* ^2^_p_ = 0.41, 1‐β = 1.00). A significant main effect of muscle region was observed for deoxy[Hb + Mb] τ_p_, which was significantly longer in VLd compared to VLs (*p* = 0.019, *η* ^2^_p_ = 0.38, 1‐β = 1.00). In addition, PHE resulted in a significant main effect of time on the change in deoxy[Hb + Mb] from 3 to 6 min (ΔA_6‐3_), which was significantly reduced in the 2nd bout (*p* < 0.001, *η* ^2^_p_ = 0.42, 1‐β = 1.00). Further, the PHE‐induced reduction in the V̇O_2SC_ was unrelated to the changes in the deoxy[Hb + Mb] slow component (A_6‐3_) in either VLs or VLd (VLs, *r* = 0.161, *p* = 0.598; VLd, *r* = 0.119, *p* = 0.699). The deoxy[Hb + Mb] τ_p_ in the 1st bout was negatively correlated with the change in deoxy[Hb + Mb] τ_p_ between the 1st and 2nd bouts in the VLs only (*r* = −0.652, *p* = 0.015; Figure 1b,c). Further, the changes in deoxy[Hb + Mb] τ_p_ between the 1st and 2nd bouts were unrelated to the changes in V̇O_2_ τ_*p*_ between the 1st and 2nd bouts in either muscle sites (VLs, *r* = −0.25, *p* = 0.40; VLd, *r* = 0.41, *p* = 0.17; Figure 5).

**TABLE 2:** The mean value of deoxy and total[Hb + Mb] concentrations during prior (1st bout) and subsequent (2nd bout) heavy cycling exercise.

	VLs		VLd		Main effect *P*		Interaction *P*
	1st	2nd	1st	2nd	Time	Site	
Deoxy[Hb + Mb] (kinetics)							
Baseline (μM)	52.5 ± 6.8	44.2 ± 7.6	50.4 ± 8.3	45.1 ± 7.7	**<0.001**	0.758	**0.040**
Amplitude (μM)	18.6 ± 7.3	29.6 ± 6.1	12.4 ± 19.2	19.3 ± 18.8	**<0.001**	0.118	**0.021**
Time delay (s)	7 ± 3	5 ± 2	6 ± 3	4 ± 3	**0.014**	0.107	0.812
Time constant (s)	9 ± 3	13 ± 3	15 ± 3	17 ± 14	0.143	**0.019**	0.885
Mean response time (s)	17 ± 3	18 ± 3	21 ± 5	21 ± 13	0.724	**0.047**	0.922
*r* ^2^	0.93 ± 0.05	0.97 ± 0.02	0.74 ± 0.24	0.76 ± 0.22			
Deoxy[Hb + Mb] (steady‐state concentrations, μM)							
Amplitude (BL‐3 min)	17.7 ± 7.8	28.9 ± 6.6	12.2 ± 18.9	19.7 ± 18.8	**<0.001**	0.155	0.051
End‐exercise (6 min)	73.7 ± 11.6	74.2 ± 11.1	66.3 ± 22.1	66 ± 21	0.800	0.211	0.413
ΔAmplitude_6‐3_ [A_6‐3_]	3.5 ± 1.8	1 ± 2.4	3.7 ± 4	1.2 ± 4.5	**0.005**	0.838	0.986
Total[Hb + Mb] (steady‐state concentrations, μM)							
Baseline	166.3 ± 13.8	171.1 ± 16.6	160.2 ± 15.9	159.7 ± 18.2	0.327	0.156	0.224
Amplitude (BL‐3 min)	17.7 ± 7.1	15.1 ± 7.8	6.3 ± 16.8	9.1 ± 15.4	0.971	0.061	0.215
End‐exercise (6 min)	183.3 ± 18.4	185.7 ± 19.7	167 ± 21	167.6 ± 22.9	0.208	**0.046**	0.458
ΔAmplitude_6‐3_ [A_6‐3_]	−0.9 ± 3.3	−0.4 ± 2	−0.1 ± 5	−1.3 ± 3.4	0.608	0.975	0.278

*Note*: Bold letters show a significant *F*‐value in two‐way ANOVA (*p* < 0.05).

### 3.3 Total[Hb + Mb] responses

**FIGURE 4:**
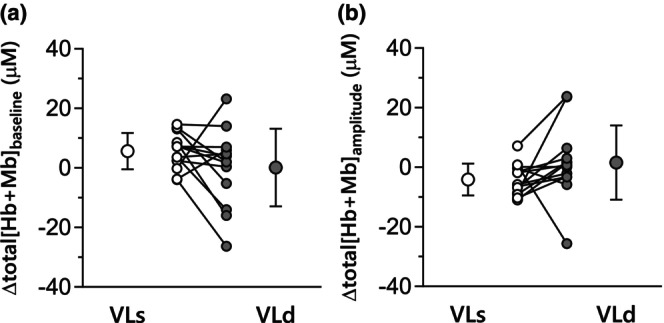
The changes in total[Hb + Mb] concentrations (baseline and amplitude) from prior to subsequent heavy‐intensity exercise in VLs (open symbol) and VLd (gray symbol). (a) Total[Hb + Mb] baseline, (b) Total[Hb + Mb] amplitude.

The group mean total[Hb + Mb] responses are presented in Table 2, Figures 2 and 4. The end‐exercise total[Hb + Mb] concentration resulted in a significant main effect of muscle region, which was lower in VLd compared with VLs (*p* = 0.046, η^2^_p_ = 0.16, 1‐β = 0.69). The change in total[Hb + Mb] from 3 to 6 min (ΔA_6‐3_) did not have any significant main effects for either time (*p* = 0.608, *η* ^2^_p_ = 0.01, 1‐β = 0.11) or muscle region (*p* = 0.975, *η* ^2^_p_ = 0.00, 1‐β = 0.05). Moreover, neither the baseline nor amplitude (i.e., baseline–3 min value) for total[Hb + Mb] had any significant main effect of time and muscle region (time effect on baseline, *p* = 0.327, *η* ^2^_p_ = 0.04, 1‐β = 0.27; time effect on amplitude, *p* = 0.971, *η* ^2^_p_ = 0.00, 1‐β = 0.05; site effect on baseline, *p* = 0.156, *η* ^2^_p_ = 0.08, 1‐β = 0.50; site effect on amplitude, *p* = 0.061, *η* ^2^_p_ = 0.14, 1‐β = 0.76; Table 2, Figure 4).

## 4 DISCUSSION

As expected, PHE led to a higher baseline pulmonary V̇O_2_ and a lower V̇O_2SC_. Within the VL, at both superficial (VLs) and deep (VLd) sites, PHE reduced the baseline deoxy[Hb + Mb] and increased its amplitude. However, there were two significant differences between VLs and VLd. Firstly, the change in both baseline and amplitude between the two exercise bouts was significantly lower in VLd compared to VLs. Moreover, the increase in the primary component time constant of deoxy[Hb + Mb] was more pronounced in VLs than VLd. Additionally, the end‐exercise total[Hb + Mb] in VLd was lower than that in VLs during both bouts.

These findings collectively suggest that the effects of PHE on microvascular Q̇O_2_‐to‐V̇O_2_ during subsequent heavy exercise are less pronounced in deep compared with superficial muscle, an effect attributable to more effective Q̇O_2_‐to‐V̇O_2_ matching in VLd under control (i.e., non‐primed) conditions. However, it is important to note that the changes in deoxy[Hb + Mb] and total[Hb + Mb] induced by PHE in either VLs or VLd were not correlated with the reduction of the slow component of V̇O_2_. Thus, it appears that the local alterations in deoxy[Hb + Mb] and total[Hb + Mb] measured herein did not contribute, at least in any quantitatively linear manner, to the reduction in the V̇O_2SC_ following PHE.

### 4.1 Priming exercise and V̇O_2_ kinetics

As previously observed (Bailey et al., 2009; Burnley et al., 2000; Fukuoka et al., 2015; Goulding et al., 2020; Saitoh et al., 2009), we found that PHE in the upright position did not affect the speed of the primary component V̇O_2_ kinetics (τ_p_). This aligns with research showing that, in young healthy individuals with τ_*p*_ values <25 s under non‐primed conditions, PHE typically does not reduce τ_*p*_, and any increases in bulk Q̇O_2_ kinetics induced by PHE are ineffectual with respect to impacting the speed of the primary component V̇O_2_ kinetics (Goulding et al., 2023). For upright heavy exercise in young healthy individuals exercising at sea‐level, this finding supports the idea that the bulk Q̇O_2_ lies to the right of the tipping point below which τ_p_ is slowed in proportion to reduced Q̇O_2_ (Korzeniewski, 2023; Poole & Jones, 2012).

### 4.2 Priming exercise and deoxy[Hb + Mb]

Examining the effects of PHE on deoxy[Hb + Mb], we observed lower baseline concentrations and greater amplitudes of deoxygenation during subsequent heavy exercise, regardless of muscle region. This suggests that the increase in microvascular perfusive Q̇O_2_ emanating from PHE is not *isolated* to a particular muscle depth within the regions examined by NIRS in our study. However, evidence of heterogeneity in intramuscular O_2_ transport strategies was found, with the reduction in deoxy[Hb + Mb] baseline and increases in deoxy[Hb + Mb] amplitude being significantly less pronounced in VLd compared with VLs. In the context of heavy‐intensity exercise, which involves recruitment of a greater population of type II muscle fibers compared to moderate‐intensity exercise (Gollnick et al., 1974; Krustrup et al., 2004; Marinari et al., 2025), there is a heightened potential for limitations in Q̇O_2_. This is due to the lower microvascular partial pressure of oxygen (PO_2_) observed in type II muscle fibers compared to their type I counterparts (McDonough et al., 2005). The deep region within the quadriceps femoris primarily consists of a higher proportion of type I fibers (Johnson et al., 1973). However, it is essential to recognize that human muscles exhibit a mosaic‐like fiber type composition, in contrast to animals with predominantly single or more stratified fiber type distribution (Delp & Duan, 1996; Saltin & Gollnick, 2011).

Other possible mechanisms for intramuscular differences (regional heterogeneity) of O_2_ transport and metabolic control properties include differential changes in sympathetic nerve activity and vasodilatory substances induced by PHE in VLs versus VLd. Specifically, during exercise, there is an accumulation of various metabolites, including hydrogen ions ([H^+^]), lactate, adenosine, and potassium ions ([K^+^]), especially in type II muscle fibers (Burnley et al., 2000; Burnley & Baker, 2005). These metabolites may facilitate greater Hb‐O_2_‐offloading (i.e., rightward shift of the Hb‐O_2_ dissociation curve) (Fukuoka et al., 2015; Gerbino et al., 1996; Jones et al., 2011; Poole et al., 1991). Additionally, the combination of centrally‐mediated reductions in sympathetic nerve activity and compromised signal transduction, along with activation of histamine H_1_ and H_2_ receptors, contribute to localized vasodilation during the post‐exercise recovery period (DeLorey & Clifford, 2022; Halliwill et al., 2013) and thus, potentially, the effects of PHE. Vascular regulation differs between fast‐ and slow‐twitch muscles (Lambert & Thomas, 2005) and may encompass variations in vascular innervation or the activity of sympathetic nerves, differences in the densities or functional reserves of vascular α‐adrenoceptors, or variations in the local production of muscle metabolites. These complex factors collectively contribute to the nuanced regulation of blood flow and O_2_ delivery within muscle tissue. Consequently, the lower reductions observed in deoxy[Hb + Mb] baseline levels from prior to subsequent exercise in VLd, as compared to VLs, suggest that the post‐exercise hyperemia effects (Hernandez et al., 2010; Koppo & Bouckaert, 2001; Poole et al., 1991) are less pronounced in VLd. This disparity might therefore be attributed to the distinct integrative regulations of sympathetic nerve activity and vasodilator substances between these two muscle regions.

Our findings relating to the time constants of deoxy[Hb + Mb] (τ) induced by PHE were intriguing, with a negative relationship between the 1st‐bout τ and changes in τ from prior to subsequent exercise in the VLs but not the VLd. Supporting this, McDonough et al. (2005) suggested that V̇O_2_ on‐kinetics in muscles with lower oxidative capacity may be hindered by insufficient oxygen availability. Hence, such muscles stand to benefit from a PHE‐induced increase in microvascular Q̇O_2_‐to‐V̇O_2_, likely accounting for why PHE‐induced changes were more pronounced in VLs than in VLd. This is further supported by the finding that the deoxy[Hb + Mb] (*τ*) was significantly longer in VLd compared to VLs, irrespective of PHE. Accordingly, following PHE, large inter‐individual differences in the slowing of muscle deoxygenation kinetics were observed in VLs, where capillary density and mitochondrial enzyme activity vary greatly depending on subjects' physiological characteristics, underscoring that PHE's impact on microvascular Q̇O_2_‐to‐V̇O_2_ is attenuated in deep compared with superficial muscle. Nonetheless, two observations indicate that VLd may still benefit from PHE: first, deoxy[Hb + Mb] baseline decreased without a change in total[Hb + Mb], suggesting improved perfusive O_2_ delivery; second, the slow component of deoxy[Hb + Mb] was attenuated in both VLd and VLs, consistent with an O_2_‐delivery‐mediated effect. Thus, while the influence of PHE is clearly greater in superficial regions, the evidence suggests that deep muscle retains some capacity to benefit from PHE by improving O_2_ supply.

**FIGURE 5:**
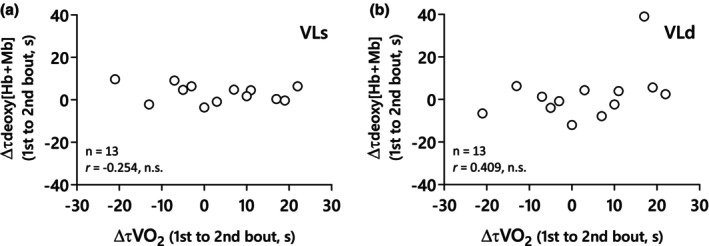
The relationship of the changes from prior to subsequent exercise between V̇O_2_ time constants and deoxy[Hb + Mb] time constants. (a) Deoxy[Hb + Mb] time constant in VLs, (b) Deoxy[Hb + Mb] time constant in VLd.

There was no relationship between the changes from prior to subsequent heavy exercise in the V̇O_2_ and deoxy[Hb + Mb] time constants at either muscle site (Figure 5), suggesting that changes in the speed of muscle deoxygenation following priming exercise are unrelated to that of the pulmonary V̇O_2_ kinetics. However, as mentioned previously, we did find an inverse relationship between τ_p_ (V̇O_2_) in the 1st bout and the changes in τ_p_ (V̇O_2_) between the 1st and 2nd bouts (Figure 1a). Examination of this relationship reveals that 5 of the 13 subjects evidenced a slowing of the primary phase V̇O_2_ kinetics following PHE (Figure 1a). This finding is surprising given the changes in mitochondrial enzyme activity (Behnke et al., 2002; Christensen et al., 2016; DeLorey et al., 2007; Gandra et al., 2012; Gurd et al., 2006; Hogan, 2001), bulk and local Q̇O_2_ (DeLorey et al., 2007; Fukuoka et al., 2015; Gerbino et al., 1996; Hernandez et al., 2010; MacDonald et al., 2001), a rightward HbO_2_‐shift (Fukuoka et al., 2015; Gerbino et al., 1996; Jones et al., 2011; Poole et al., 1991), and altered motor unit recruitment patterns (Burnley et al., 2000; Burnley & Baker, 2005) known to occur with PHE and each of these changes would be expected to speed V̇O_2_ kinetics. This expectation is consistent with previous findings by Goulding et al., (2020), wherein the change of the speed of V̇O_2_ kinetics induced by hyperoxic breathing was inversely related to the value of τp (V̇O_2_) in normoxia. Hence, subjects with initially rapid V̇O_2_ kinetics in normoxia became slower in hyperoxia and subjects with sluggish V̇O_2_ kinetics in normoxia became faster in hyperoxia. One interpretation of this finding is that in subjects with initially rapid τ_p_ values (i.e., <25 s), the rates of change in muscle Q̇O_2_ and V̇O_2_ are well‐matched at the onset of exercise. When any intervention is imposed that increases the rate of Q̇O_2_ above that of V̇O_2_, the fall in venous O_2_ content at the onset of subsequent exercise *is lessened*, resulting in a slowing of pulmonary (but not muscle) V̇O_2_ kinetics (Benson et al., 2013). Hence, it is possible that a dissociation between muscle and pulmonary V̇O_2_ could explain this finding. Alternatively, it was recently shown by Grassi et al. (2023) that hyperoxia induced a slowing of muscle V̇O_2_ kinetics in oxidative canine hindlimb muscle suggesting that, in addition to a dissociation between muscle and lung V̇O_2_, a direct slowing of muscle V̇O_2_ kinetics, perhaps due to increased intracellular (PCr) buffering of the rise in (ADP), could also contribute to the findings displayed in Figure 1.

### 4.3 Priming exercise and total[Hb + Mb]

A novel finding herein was that PHE did not induce an increase in total[Hb + Mb] baseline levels in either VLs or VLd and therefore could not help explain the reduced V̇O_2SC_. Muscle diffusive O_2_ conductance is primarily determined by the aggregate number of red blood cells adjacent to the contracting myocytes (Federspiel & Popel, 1986), hence changes in total [Hb + Mb] measured via NIRS during exercise are reflective of changes in the vascular component of diffusive O_2_ conductance (Grassi et al., 2019). Thus, increased diffusive O_2_ conductance of these particular local muscle regions is not obligatory for a faster overall pulmonary V̇O_2_ kinetics (i.e., reduction of the slow components) after PHE.

It was noteworthy that the end‐exercise total[Hb + Mb] concentration in VLd was consistently lower than in VLs for both the 1st and 2nd bouts of exercise (Table 2). However, as there was no PHE‐induced change in either muscle site, there was a greater emphasis on perfusive rather than diffusive O_2_ transport in VLd. Somewhat differently, Goulding et al. (2020) reported that, following PHE, both baseline and end‐exercise total[Hb + Mb] were increased in both in the VLs and VLd during exercise. Additionally, Fukuoka et al. (Fukuoka et al., 2015) found that the PHE‐induced increase in total[Hb + Mb] at baseline and end‐exercise across the superficial muscle sites was associated with the reduced V̇O_2SC_, possibly consequent to an augmented muscle O_2_ diffusing capacity (Federspiel & Popel, 1986; Groebe & Thews, 1990) and its intracellular consequences (i.e., elevated intramyocyte PO_2_‐induced enhancement of mitochondrial oxidative regulation). As above, the precise mechanistic bases for these disparities between studies present and past (Hernandez et al., 2010; Poole et al., 1991) may be attributed to differences among participants in subjects' training status, fitness, and any associated variation of muscle structure (i.e., fiber type and vascular density) and/or function (i.e., fiber activation, vascular, blood flow and metabolic control) and are the topic of ongoing investigation. Although we originally hypothesized that perfusion‐metabolism matching might be superior in VLd under unprimed conditions due to its oxidative phenotype, our data did not reveal significant differences in deoxy[Hb + Mb] kinetics between VLs and VLd in Bout 1. Therefore, we cannot conclusively attribute the smaller priming‐induced changes in muscle deoxygenation dynamics in VLd to baseline advantages in Q̇O_2_‐to‐V̇O_2_ matching. These interpretations should be considered speculative and warrant further investigation.

One potential limitation of the present study is the performance of exercise at 50%Δ, which approximates the heavy‐severe border and raises the possibility that some subjects were exercising in different intensity domains (Keir et al., 2015). Moreover, methodological limitations relating to the assessment of deep muscle tissue must be considered. With the 6 cm optode spacing used to target the deep muscle regions in this study, photon paths inevitably traverse superficial tissues, resulting in signal contamination. Therefore, it cannot be excluded that some of the results interpreted as reflecting deep muscle potential may have been influenced by superficial tissue signals. Accordingly, interpretation of the deep muscle findings requires appropriate caution.

### 4.4 Perspectives and significance

This investigation supports that: (1) PHE increases microvascular Q̇O_2_‐to‐V̇O_2_ during subsequent heavy exercise in deep muscle to a lesser degree compared to superficial muscle; (2) the lower total[Hb + Mb] in VLd compared to VLs reflects reduced reliance on diffusive O_2_ transport in deep muscle; and (3) despite site‐dependent differences in PHE‐induced alterations in microvascular Q̇O_2_‐to‐V̇O_2_ between regions, these changes in either muscle region did not contribute to a reduction in the V̇O_2SC_ or alterations in the speed of the primary component kinetics. Accordingly, PHE induced a relatively greater reliance on perfusive Q̇O_2_ rather than diffusive O_2_ transport in deep versus superficial muscle during the subsequent heavy exercise. The present investigation lays valuable groundwork for construction and testing of hypotheses for *future studies* in healthy aging and patient populations where different Q̇O_2_‐to‐V̇O_2_ *matching* in superficial versus deep muscle may be linked to exercise intolerance.

## AUTHOR CONTRIBUTIONS

W.C., D.O., and S.K. conception and design of research. W.C., D.O. and S.K. performed experiments. W.C., D.O., R.P.G., D.C.P., and S.K. analyzed data. W.C., D.O., R.P.G., D.C.P., T.J.B., and S.K. interpreted results of experiments. D.O. and S.K. prepared figures. W.C., D.O., R.P.G., D.C.P., T.J.B., and S.K. drafted manuscript. W.C., D.O., R.P.G., D.C.P., T.J.B., A.M.J., P.‐J.M., N.K., and S.K. edited and revised manuscript. W.C., D.O., R.P.G., D.C.P., T.J.B., A.M.J., P.‐J.M., N.K., and S.K. approved final version of manuscript.

## FUNDING INFORMATION

Weerapong Chidnok was supported by Naresuan University (5/2566), Thailand. S. Koga was supported by grants from Japan Society for the Promotion of Science (KAKENHI‐16K13011).

## CONFLICT OF INTEREST STATEMENT

The authors declare no conflicts of interest.

## Data Availability

Data will be made available upon reasonable request.
